# “Miles to go before I seek”: distance to the health facility and health care use among older adults in India

**DOI:** 10.1016/j.lansea.2025.100579

**Published:** 2025-04-16

**Authors:** Sheuli Misra, Jeetendra Yadav, Abhinav Sinha, Krushna Chandra Sahoo, Shweta Tanwar, Sneh Shalini, Arohi Chauhan, Sanghamitra Pati

**Affiliations:** a WHO Country Office for India, R K Khanna Tennis Stadium, Africa Avenue, New Delhi, 110029, India; b ICMR-National Institute for Research in Digital Health and Data Science New Delhi, India; c Academy of Scientific and Innovative Research (AcSIR), Kamla Nehru Nagar, Ghaziabad, Uttar Pradesh, 201002, India; d ICMR-Regional Medical Research Centre, Bhubaneswar, Odisha, 751023, India; e Health Technology Assessment in India (HTAIn), Department of Health Research, Ministry of Health & Family Welfare, Govt. of India, India; f Indian Council of Medical Research, Ansari Nagar, New Delhi, India; g South Asian Institute of Health Promotion, India

India, home to 138 million older adults, is witnessing a rapid demographic transition, with the proportion of the population aged 60 and above increasing from 7.4% in 2001 to a projected 13.2% in 2031.^1^ Nearly half of these population have multiple long-term or debilitating conditions that demands continuous and coordinated health care.^2^ Further, the complexity of healthcare requirements increases as individuals age, necessitating frequent and urgent medical attention.^3^ However, access to such care is often restricted by factors such as the availability of local health services, financial constraints, low health literacy, and inadequate family or social support systems.^4^ While much research has explored financial constraints and health literacy as barriers to healthcare access, there is limited evidence on how physical distance impacts healthcare utilization and health-seeking behaviours in older adults in India. We used the nationally representative Longitudinal Ageing Study of India (LASI) (Wave-1, 2017–18) consisting of 31,902 older adults’ data to analyse the average distance travelled by older adults for their routine and acute health care needs and concomitant health care utilization through an equity lens.^5^

**Table 1:** Healthcare utilization patterns among elderly (60+) by distance to health facility and socioeconomic characteristics, LASI-India 2017–2018.

Disease categories	Inpatient					Outpatient				
	0–10	11–30	31–60	61–120	120+	0–10	11–30	31–60	61–120	120+
**Sex**										
Male	35.7	30.2	16.7	9.5	8.0	70.5	17.5	6.5	4.2	1.3
Female	45.8	26.8	12.8	7.8	6.8	75.4	16.7	4.8	2.4	0.6
**Marital status**										
Currently married	37.9	28.5	15.5	10.3	7.8	70.8	17.8	6.3	3.9	1.1
Others^a^	45.8	28.3	13.3	5.7	6.8	76.8	15.9	4.3	2.3	0.7
**Place of residence**										
Rural	28.3	33.2	19.6	10.6	8.3	67.1	21.2	7.1	3.7	0.9
Urban	67.7	18.3	4.1	4.5	5.4	87.2	7.3	1.9	2.4	1.2
**Living arrangements**										
Living alone	60.0	18.2	10.8	5.2	5.8	75.4	15.5	5.9	2.6	0.5
With spouse and/or others	35.9	27.7	18.6	10.7	7.2	68.2	20.7	6.7	3.2	1.2
With spouse and children	39.0	29.1	13.9	9.9	8.1	72.1	16.4	6.1	4.2	1.1
With children and others	42.6	30.8	13.0	6.5	7.2	76.1	16.5	4.3	2.3	0.7
Living with others only	43.5	28.3	19.9	3.3	5.0	81.8	12.9	2.7	2.1	0.6
**Education status**										
No education	40.6	28.3	15.0	8.7	7.4	72.8	18.6	5.7	2.4	0.6
Primary	45.4	26.7	13.7	8.8	5.3	72.5	16.4	5.5	4.6	1.0
Secondary	31.0	36.0	16.7	9.1	7.3	74.2	15.2	5.3	3.6	1.7
Higher secondary & above	41.6	21.9	13.3	6.3	17.0	75.7	11.4	5.4	5.0	2.5
**Wealth quintile**										
Poorest	45.9	28.1	12.5	5.3	8.1	80.0	12.6	4.1	2.5	0.8
Poorer	46.0	23.4	17.4	7.2	6.0	76.6	15.1	5.1	2.6	0.7
Middle	38.4	30.7	13.0	11.3	6.6	74.3	17.9	4.5	2.3	1.0
Richer	43.2	29.6	14.0	6.8	6.4	68.2	20.3	7.0	3.8	0.8
Richest	34.1	29.8	15.8	11.0	9.3	65.7	19.7	7.4	5.6	1.6
**Employment**										
Currently not working/receiving pension	38.4	29.0	16.0	9.2	7.4	71.9	18.4	5.4	3.4	0.9
Formal	22.7	33.7	23.2	12.0	8.4	66.1	20.9	8.6	3.9	0.5
Informal	35.4	30.7	15.7	8.7	9.6	72.2	15.5	8.0	3.8	0.6
Pensioners	40.9	22.1	16.8	8.1	12.2	72.4	13.3	6.1	5.5	2.7
**Type of health facility**										
Public	46.4	27.8	13.5	7.0	5.3	45.4	24.0	17.1	9.5	4.0
Private	37.4	28.9	15.5	9.7	8.7	39.4	29.8	14.6	8.7	7.5
**Health insurance**										
Yes	38.2	28.9	16.4	7.7	8.9	70.7	18.7	5.9	3.4	1.4
No	41.5	28.4	14.3	9.0	7.0	73.7	16.7	5.5	3.3	0.9
**Region**										
North	42.2	25.5	13.9	12.2	6.2	68.6	20.2	6.3	3.8	1.0
Central	44.7	24.8	14.8	9.5	6.2	75.1	14.5	6.0	3.9	0.5
East	40.0	33.0	11.4	6.8	8.8	77.1	14.8	4.7	2.1	1.3
Northeast	49.9	15.4	13.7	10.4	10.5	71.0	16.4	5.2	5.0	2.4
West	40.2	32.4	12.2	6.4	8.9	74.5	16.5	4.8	3.0	1.1
South	39.9	34.2	15.6	5.5	4.8	68.9	22.2	5.7	2.6	0.7
**States**										
Andhra Pradesh	29.8	21.4	30.3	10.0	8.6	60.3	22.4	11.0	3.6	2.7
Arunachal Pradesh	45.9	23.8	1.9	14.7	13.8	32.4	30.7	1.3	10.5	25.0
Assam	48.9	5.5	28.7	9.2	7.8	70.4	14.3	6.3	7.0	2.0
Bihar	30.9	27.8	20.4	7.2	13.7	77.2	14.3	5.2	3.3	0.0
Chhattisgarh	29.3	37.8	8.0	19.6	5.4	71.3	19.8	2.4	5.2	1.4
Goa	49.5	36.7	10.2	2.6	1.0	66.8	27.8	4.8	0.5	0.0
Gujarat	42.2	38.3	4.6	7.2	7.6	76.5	13.6	5.4	4.5	0.1
Haryana	40.0	37.6	9.3	6.1	7.1	71.9	21.0	4.8	2.4	0.0
Himachal Pradesh	4.5	27.4	29.6	20.7	17.8	49.5	21.5	13.6	10.0	5.5
Jharkhand	30.7	31.6	10.9	14.7	12.1	63.2	22.1	6.7	2.9	5.1
Karnataka	39.9	23.1	15.5	14.2	7.3	75.6	17.3	2.7	4.4	0.0
Kerala	59.3	36.1	3.8	0.3	0.6	83.7	14.4	1.4	0.5	0.0
Madhya Pradesh	49.8	25.1	16.4	6.0	2.7	57.2	23.5	15.2	4.1	0.0
Maharashtra	38.8	28.9	14.1	7.9	10.2	73.7	17.0	5.1	3.1	1.1
Manipur	74.7	10.5	5.4	6.8	2.6	78.4	13.2	4.8	3.1	0.4
Meghalaya	46.6	27.4	19.0	7.0	0.0	65.6	16.9	5.3	10.7	1.4
Mizoram	31.5	3.2	14.4	12.8	38.1	57.2	9.2	14.4	8.5	10.8
Nagaland	0.0	78.4	0.1	7.4	14.0	36.1	35.9	11.5	5.7	10.7
Odisha	33.6	20.9	12.3	22.8	10.4	74.2	13.5	5.6	3.2	3.5
Punjab	46.0	31.1	13.0	9.9	0.0	75.9	16.1	4.5	3.0	0.5
Rajasthan	38.5	21.2	18.0	15.2	7.2	68.8	19.0	7.2	4.7	0.4
Sikkim	17.6	26.9	25.5	25.4	4.6	56.1	19.4	17.3	4.9	2.3
Tamil Nadu	39.3	32.3	20.2	3.6	4.6	75.8	18.7	4.4	0.9	0.2
Telangana	35.2	29.7	17.6	8.9	8.6	49.2	35.2	9.5	4.5	1.7
Tripura	80.4	6.4	10.6	0.0	2.6	87.9	5.9	2.8	1.6	1.9
Uttar Pradesh	38.7	26.1	15.3	9.4	10.5	77.4	12.7	5.1	4.1	0.7
Uttarakhand	29.2	19.9	14.7	20.1	16.2	60.4	23.8	7.1	4.5	4.3
West Bengal	47.4	35.3	8.9	4.9	3.6	80.7	14.6	2.6	0.6	1.5
**Union Territories (UTs)**										
Andaman and Nicobar	36.4	57.0	0.0	0.2	6.4	66.7	31.3	0.0	0.8	1.3
Chandigarh	72.0	24.7	0.0	3.3	0.0	91.9	7.1	0.9	0.0	0.1
Dadra and Nagar Haveli	55.7	22.9	7.6	3.5	10.4	82.0	15.5	1.3	1.2	0.0
Daman and Diu	45.8	31.4	4.3	10.3	8.3	69.5	15.0	5.8	9.7	0.0
Jammu and Kashmir	50.1	11.7	17.0	12.4	8.8	53.5	32.7	6.2	4.9	2.8
Lakshadweep	64.8	0.0	0.9	2.4	31.9	85.9	0.8	0.0	0.5	12.8
Puducherry	56.8	15.1	7.7	4.6	15.8	92.0	6.1	0.9	0.7	0.4
Delhi	85.9	11.8	2.3	0.0	0.0	91.7	8.3	0.0	0.0	0.0
**Total**	**40.7**	**28.5**	**14.8**	**8.7**	**7.4**	**73.1**	**17.1**	**5.6**	**3.3**	**1.0**

a Includes widowed/divorced/separated/others.

Older adults, on an average travelled a distance of nine miles (14.54 km) to seek outpatient services and 27 miles (43.62 km) for inpatient care respectively. Moreover, for two thirds (67%) of the urban older adults, the availed outpatient facility was within six miles (10 km) of reach and for rural counterparts the same was 17.5 miles (28.3 km), displaying significant urban-rural disparity. This grew disproportionately for in-patient care where the distance and time taken was two times higher for rural sexagenarian than their urban counterparts. For in-patient admission, 95 percent arranged their own mode of transport, while five percent used ambulance services, with no significant urban-rural difference.

Both out-patient and in-patient care utilization was high (73% and 40% respectively) when the facility distance was within 10 km. As the distance increased, a commensurate decline in the out-patient utilization was observed being 17% and 10% for facilities at a distance of 11–30 km and 30 km or more respectively. Additionally, for women, those living alone, and with low education and income, this decline was more pronounced. Around 19% of rural older adults had to travel at least 60 km to avail in-patient care. The situation was similar for urban dwellers with 10% travelling at least 60 km for in-patient care.

The state-wise data highlights that older people in Kerala (59%), Tripura (80.4%) and Manipur (74.7%) had more inpatient visits within 0–10 km. While in Kerala it could be attributed to easy access and better availability of health infrastructure, in Manipur and Tripura it may be because people rely on nearby facilities during emergency inpatient situations. Potential geographical challenges are also evident in hilly states like Nagaland (0%), Sikkim (17.6%), and Himachal Pradesh (4.5%) which show fewer inpatient visits within 0–10 km. Moreover, higher percentages of inpatient visits at distances over 61 km in the state of Mizoram (51%), Nagaland (21.5%), and Himachal Pradesh (38.5%), indicating poor and limited accessibility to nearby health facilities. The majority outpatient visits are within 0–10 km in most states, with the highest in Tripura (87.9%), Kerala (83.7%) and Manipur (78.4%). However, many northeastern states show a lower percentage of outpatient visits within 0–10 km and a higher percentage beyond >61 km, indicating that older individuals often travel farther for outpatient care due to unavailability of nearby outpatient health facilities. **Uttar Pradesh, Bihar, and Madhya Pradesh** show moderate proximity (11–60 km) for outpatient care but higher share of inpatient visits at distances beyond 30 km. The less variability in distance among southern states indicates well-distributed healthcare infrastructure. While higher reliance on distant facilities among northeastern and hilly states of north India indicates potential need for better healthcare infrastructure at the local level. Across UTs Jammu and Kashmir, Daman and Diu, and Lakshadweep show older people from these UTs travel very long for any healthcare visits (both inpatient and outpatient) indicating extremely limited availability of local health facilities (Table 1).

Our analysis demonstrated significant variations in health facility distance and the implied travel burden among older adults. Longer travel time and farther facility act as a potential barrier for receiving timely and essential healthcare for this population which could posit higher risk of adverse outcomes. Addressing transportation barriers appears to be one of the key strategies for improving access to care among geriatric population, especially those residing in rural areas. Various studies have shown that interventions aimed at minimizing transportation barriers among low-income, remote and older population not only improves access to medical care but patient outcome as well, while being cost-efficient. Future research must develop and demonstrate how community-based transport service can be embedded within as a model for implementation for geriatric care. Given the rising number of ageing populations who are home-bound, a shift from clinic-based out-patient care to home-based primary care merits consideration through a mix of mobile medical van, digital healthcare and inclusive social support. There is a need to design and formulate strategies on how existing Ayushman Arogya Mandir (Community based primary care centre) can be strengthened to meet the comprehensive healthcare needs of growing geriatric population. Given the evidence of longer distance to avail the in-patient services, the district health system (secondary healthcare) must also be equipped with necessary specialized care to avoid undue referral. This would further reduce the healthcare travel burden and improve healthcare utilization among older adults and promote healthy longevity.

Our findings revealed that even in universal healthcare system, there continues to remain marked disparities in access to care among older adults with distance to the facility as a critical barrier. Ensuring availability of health services within reach and reducing geographical barriers are paramount towards an equitable and inclusive healthcare system that ensures no one is left behind.

## Contributors

**SM:** Methodology, Analysis, Data Curation, Writing-draft, and Editing**; JY:** Methodology, Analysis, Supervision, Validation, Data Curation, Writing review and Editing; **AVN:** Writing-original draft, Validation, Writing review and Editing; **KCS:** Methodology, Analysis, Visualization, Writing-original draft, Writing review and Editing; **ST**: Methodology, Visualization, Writing-original draft, Writing review and Editing; **SS:** Supervision, Writing review and Editing; **AP:** Writing review and Editing **SP:** Conceptualization, Supervision, Validation, Writing review and Editing.

## Data sharing statement

This study utilised the LASI wave 1 round data, which is accessible to individuals upon request.

## Declaration of interests

The authors declare no conflict of interest.
