# Corrigendum to ““Miles to go before I seek”: distance to the health facility and health care use among older adults in India” [The Lancet Regional Health Southeast Asia. Volume 37, 100579, April 2025]

**DOI:** 10.1016/j.lansea.2025.100702

**Published:** 2025-12-23

**Authors:** Sheuli Misra, Jeetendra Yadav, Abhinav Sinha, Krushna Chandra Sahoo, Shweta Tanwar, Sneh Shalini, Arohi Chauhan, Sanghamitra Pati

**Affiliations:** a WHO Country Office for India, R K Khanna Tennis Stadium, Africa Avenue, New Delhi, 110029, India; b ICMR-National Institute for Research in Digital Health and Data Science New Delhi, India; c Academy of Scientific and Innovative Research (AcSIR), Kamla Nehru Nagar, Ghaziabad, Uttar Pradesh, 201002, India; d ICMR-Regional Medical Research Centre, Bhubaneswar, Odisha, 751023, India; e Health Technology Assessment in India (HTAIn), Department of Health Research, Ministry of Health & Family Welfare, Govt. of India, India; f Indian Council of Medical Research, Ansari Nagar, New Delhi, India; g South Asian Institute of Health Promotion, India

## Correction 1

The following corrections are to be made in the main text:

• The interpretation of the sentence “Moreover, for two thirds (67%) of the urban older adults, the availed outpatient facility was within six miles (10 km) of reach and for rural counterparts the same was 17.5 miles (28.3 km), displaying significant urban-rural disparity” to be corrected as “Moreover, for two thirds (68%) of the urban older adults availed outpatient services within six miles (10 km), whereas their rural counterparts had to travel 17.5 miles (28.3 km), displaying a significant urban-rural disparity.”
• The interpretation presented in the paragraph “Both out-patient and in-patient care utilization was high (73% and 40% respectively) when the facility distance was within 10 km. As the distance increased, a commensurate decline in the out-patient utilization was observed being 17% and 10% for facilities at a distance of 11–30 km and 30 km or more respectively. Additionally, for women, those living alone, and with low education and income, this decline was more pronounced. Around 19% of rural older adults had to travel at least 60 km to avail in-patient care. The situation was similar for urban dwellers with 10% travelling at least 60 km for in-patient care” is to be corrected as “A higher proportion of elderly travelled between 0 and 10 km for in-patient and out-patient healthcare services (41% and 73%, respectively). As the distance increased, a commensurate decline in the out-patient utilization was observed, with 17% of the elderly travelling 11–30 km and only 10% travelled a distance of 30 km or more. This decline was more pronounced among elderly women, those living alone, and with low education and income. Around 19% of rural older adults and 10% of urban older adults travelled at least 60 km to avail in-patient care.”
• The interpretation of the sentence “The state-wise data highlights that older people in Kerala (59%), Tripura (80.4%) and Manipur (74.7%) had more inpatient visits within 0–10 km” is to be corrected as “The state-wise data highlights that a higher proportion of the older population in Kerala (59%), Tripura (80.4%) and Manipur (74.7%) travelled between 0 and 10 km for inpatient care.”
• The interpretation of the sentence “Potential geographical challenges are also evident in hilly states like Nagaland (0%), Sikkim (17.6%), and Himachal Pradesh (4.5%) which show fewer inpatient visits within 0–10 km” is to be corrected as “Potential geographical challenges are particularly evident in hilly states like Nagaland (0%), Sikkim (17.6%), and Himachal Pradesh (4.5%), which show a lower proportion of older individuals seeking inpatient visits within 0–10 km”.
• The interpretation of the sentence “Moreover, higher percentages of inpatient visits at distances over 61 km in the state of Mizoram (51%), Nagaland (21.5%), and Himachal Pradesh (38.5%), indicating poor and limited accessibility to nearby health facilities” is to be corrected as “Moreover, a significant share of older adults in the state of Mizoram (51%), Nagaland (21.5%), and Himachal Pradesh (38.5%), travelled over 61 km for inpatient visits, indicating poor and limited accessibility to nearby health facilities.”
• The interpretation of the sentence “The majority outpatient visits are within 0–10 km in most states, with the highest in Tripura (87.9%), Kerala (83.7%) and Manipur (78.4%)” is to be corrected as “The majority older adults sought outpatient care within 0–10 km in most states, with the highest share in Tripura (87.9%), Kerala (83.7%) and Manipur (78.4%).”
• The interpretation of the sentence “However, many northeastern states show a lower percentage of outpatient visits within 0–10 km and a higher percentage beyond >61 km, indicating that older individuals often travel farther for outpatient care due to unavailability of nearby outpatient health facilities” is to be corrected as “However, a higher percentage of elderly specifically in many northeastern states travelled beyond >61 km, indicating unavailability of nearby outpatient health facilities.”
• The interpretation of the sentence “Uttar Pradesh, Bihar, and Madhya Pradesh show moderate proximity (11–60 km) for outpatient care but higher share of inpatient visits at distances beyond 30 km” is to be corrected as “Older people residing in Uttar Pradesh, Bihar, and Madhya Pradesh often travelled between 11 and 60 km for outpatient care while a larger share travelled beyond 30 km for inpatient visits.”
• The interpretation of the sentence “Across UTs Jammu and Kashmir, Daman and Diu, and Lakshadweep show older people from these UTs travel very long for any healthcare visits (both inpatient and outpatient) indicating extremely limited availability of local health facilities” is to be corrected as “Across UTs a higher share of older people from Dadra and Nagar Haveli, Lakshadweep, and Puducherry has to travel very long (>120 km) for inpatient health services, indicating limited availability of state-of-art health facilities for geriatric care.”

## Correction 2

There was an inadvertent issue in Table 1 where we wrote “Disease categories” as heading for the first column. We have not presented any empirics or interpretation by disease categories. We sincerely regret for this oversight.

**Table 1:** Proportion of elderly (60+) who recently visited inpatient and outpatient health services, categorized by distance travelled and socioeconomic characteristics, LASI-India 2017–2018.

	Inpatient					Outpatient				
	0–10	11–30	31–60	61–120	120+	0–10	11–30	31–60	61–120	120+
**Sex**										
Male	35.7	30.2	16.7	9.5	8.0	70.5	17.5	6.5	4.2	1.3
Female	45.8	26.8	12.8	7.8	6.8	75.4	16.7	4.8	2.4	0.6
**Marital status**										
Currently married	37.9	28.5	15.5	10.3	7.8	70.8	17.8	6.3	3.9	1.1
Others^a^	45.8	28.3	13.3	5.7	6.8	76.8	15.9	4.3	2.3	0.7
**Place of residence**										
Rural	28.3	33.2	19.6	10.6	8.3	67.1	21.2	7.1	3.7	0.9
Urban	67.7	18.3	4.1	4.5	5.4	87.2	7.3	1.9	2.4	1.2
**Living arrangements**										
Living alone	60.0	18.2	10.8	5.2	5.8	75.4	15.5	5.9	2.6	0.5
With spouse and/or others	35.9	27.7	18.6	10.7	7.2	68.2	20.7	6.7	3.2	1.2
With spouse and children	39.0	29.1	13.9	9.9	8.1	72.1	16.4	6.1	4.2	1.1
With children and others	42.6	30.8	13.0	6.5	7.2	76.1	16.5	4.3	2.3	0.7
Living with others only	43.5	28.3	19.9	3.3	5.0	81.8	12.9	2.7	2.1	0.6
**Education status**										
No education	40.6	28.3	15.0	8.7	7.4	72.8	18.6	5.7	2.4	0.6
Primary	45.4	26.7	13.7	8.8	5.3	72.5	16.4	5.5	4.6	1.0
Secondary	31.0	36.0	16.7	9.1	7.3	74.2	15.2	5.3	3.6	1.7
Higher secondary & above	41.6	21.9	13.3	6.3	17.0	75.7	11.4	5.4	5.0	2.5
**Wealth quintile**										
Poorest	45.9	28.1	12.5	5.3	8.1	80.0	12.6	4.1	2.5	0.8
Poorer	46.0	23.4	17.4	7.2	6.0	76.6	15.1	5.1	2.6	0.7
Middle	38.4	30.7	13.0	11.3	6.6	74.3	17.9	4.5	2.3	1.0
Richer	43.2	29.6	14.0	6.8	6.4	68.2	20.3	7.0	3.8	0.8
Richest	34.1	29.8	15.8	11.0	9.3	65.7	19.7	7.4	5.6	1.6
**Employment**										
Currently not working/receiving pension	38.4	29.0	16.0	9.2	7.4	71.9	18.4	5.4	3.4	0.9
Formal	22.7	33.7	23.2	12.0	8.4	66.1	20.9	8.6	3.9	0.5
Informal	35.4	30.7	15.7	8.7	9.6	72.2	15.5	8.0	3.8	0.6
Pensioners	40.9	22.1	16.8	8.1	12.2	72.4	13.3	6.1	5.5	2.7
**Type of health facility**										
Public	46.4	27.8	13.5	7.0	5.3	45.4	24.0	17.1	9.5	4.0
Private	37.4	28.9	15.5	9.7	8.7	39.4	29.8	14.6	8.7	7.5
**Health insurance**										
Yes	38.2	28.9	16.4	7.7	8.9	70.7	18.7	5.9	3.4	1.4
No	41.5	28.4	14.3	9.0	7.0	73.7	16.7	5.5	3.3	0.9
**Region**										
North	42.2	25.5	13.9	12.2	6.2	68.6	20.2	6.3	3.8	1.0
Central	44.7	24.8	14.8	9.5	6.2	75.1	14.5	6.0	3.9	0.5
East	40.0	33.0	11.4	6.8	8.8	77.1	14.8	4.7	2.1	1.3
Northeast	49.9	15.4	13.7	10.4	10.5	71.0	16.4	5.2	5.0	2.4
West	40.2	32.4	12.2	6.4	8.9	74.5	16.5	4.8	3.0	1.1
South	39.9	34.2	15.6	5.5	4.8	68.9	22.2	5.7	2.6	0.7
**States**										
Andhra Pradesh	29.8	21.4	30.3	10.0	8.6	60.3	22.4	11.0	3.6	2.7
Arunachal Pradesh	45.9	23.8	1.9	14.7	13.8	32.4	30.7	1.3	10.5	25.0
Assam	48.9	5.5	28.7	9.2	7.8	70.4	14.3	6.3	7.0	2.0
Bihar	30.9	27.8	20.4	7.2	13.7	77.2	14.3	5.2	3.3	0.0
Chhattisgarh	29.3	37.8	8.0	19.6	5.4	71.3	19.8	2.4	5.2	1.4
Goa	49.5	36.7	10.2	2.6	1.0	66.8	27.8	4.8	0.5	0.0
Gujarat	42.2	38.3	4.6	7.2	7.6	76.5	13.6	5.4	4.5	0.1
Haryana	40.0	37.6	9.3	6.1	7.1	71.9	21.0	4.8	2.4	0.0
Himachal Pradesh	4.5	27.4	29.6	20.7	17.8	49.5	21.5	13.6	10.0	5.5
Jharkhand	30.7	31.6	10.9	14.7	12.1	63.2	22.1	6.7	2.9	5.1
Karnataka	39.9	23.1	15.5	14.2	7.3	75.6	17.3	2.7	4.4	0.0
Kerala	59.3	36.1	3.8	0.3	0.6	83.7	14.4	1.4	0.5	0.0
Madhya Pradesh	49.8	25.1	16.4	6.0	2.7	57.2	23.5	15.2	4.1	0.0
Maharashtra	38.8	28.9	14.1	7.9	10.2	73.7	17.0	5.1	3.1	1.1
Manipur	74.7	10.5	5.4	6.8	2.6	78.4	13.2	4.8	3.1	0.4
Meghalaya	46.6	27.4	19.0	7.0	0.0	65.6	16.9	5.3	10.7	1.4
Mizoram	31.5	3.2	14.4	12.8	38.1	57.2	9.2	14.4	8.5	10.8
Nagaland	0.0	78.4	0.1	7.4	14.0	36.1	35.9	11.5	5.7	10.7
Odisha	33.6	20.9	12.3	22.8	10.4	74.2	13.5	5.6	3.2	3.5
Punjab	46.0	31.1	13.0	9.9	0.0	75.9	16.1	4.5	3.0	0.5
Rajasthan	38.5	21.2	18.0	15.2	7.2	68.8	19.0	7.2	4.7	0.4
Sikkim	17.6	26.9	25.5	25.4	4.6	56.1	19.4	17.3	4.9	2.3
Tamil Nadu	39.3	32.3	20.2	3.6	4.6	75.8	18.7	4.4	0.9	0.2
Telangana	35.2	29.7	17.6	8.9	8.6	49.2	35.2	9.5	4.5	1.7
Tripura	80.4	6.4	10.6	0.0	2.6	87.9	5.9	2.8	1.6	1.9
Uttar Pradesh	38.7	26.1	15.3	9.4	10.5	77.4	12.7	5.1	4.1	0.7
Uttarakhand	29.2	19.9	14.7	20.1	16.2	60.4	23.8	7.1	4.5	4.3
West Bengal	47.4	35.3	8.9	4.9	3.6	80.7	14.6	2.6	0.6	1.5
**Union Territories (UTs)**										
Andaman and Nicobar	36.4	57.0	0.0	0.2	6.4	66.7	31.3	0.0	0.8	1.3
Chandigarh	72.0	24.7	0.0	3.3	0.0	91.9	7.1	0.9	0.0	0.1
Dadra and Nagar Haveli	55.7	22.9	7.6	3.5	10.4	82.0	15.5	1.3	1.2	0.0
Daman and Diu	45.8	31.4	4.3	10.3	8.3	69.5	15.0	5.8	9.7	0.0
Jammu and Kashmir	50.1	11.7	17.0	12.4	8.8	53.5	32.7	6.2	4.9	2.8
Lakshadweep	64.8	0.0	0.9	2.4	31.9	85.9	0.8	0.0	0.5	12.8
Puducherry	56.8	15.1	7.7	4.6	15.8	92.0	6.1	0.9	0.7	0.4
Delhi	85.9	11.8	2.3	0.0	0.0	91.7	8.3	0.0	0.0	0.0
**Total**	**40.7**	**28.5**	**14.8**	**8.7**	**7.4**	**73.1**	**17.1**	**5.6**	**3.3**	**1.0**

a Includes widowed/divorced/separated/others.

## Correction 3

We recognized that the table heading “Healthcare utilization patterns among elderly (60+) by distance to health facility and socioeconomic characteristics, LASI-India 2017–2018” is to be changed to “Proportion of elderly (60+) who recently visited inpatient and outpatient health services, categorized by distance travelled and socioeconomic characteristics, LASI-India 2017–2018”, since the table shows what percentage (%) of older people has travelled a certain distance to get a health services (inpatient/outpatient).

## Correction 4

We would also like to attach the methods which is part of the Appendix.

