# Supplementary material for: Corrigendum to ““Miles to go before I seek”: distance to the health facility and health care use among older adults in India” [The Lancet Regional Health Southeast Asia. Volume 37, 100579, April 2025]
Source: Lancet Reg Health Southeast Asia. 2025 Dec 23;45:100702. doi: 10.1016/j.lansea.2025.100702 (PMC12926031; doi:10.1016/j.lansea.2025.100702)
Supplement: Methods [file mmc1.docx]

**Methods**

We analysed the first-wave data of the Longitudinal Ageing Study of India (LASI), conducted between 2017-2018, which interviewed individuals aged 45 and above (including their partners, irrespective of age) across 28 states and 8 union territories (UTs) of India. The section I_D. Health Care Access and Utilization (HC) of the LASI individual questionnaire has collected information on individuals’ visits to any health facilities in the past 12 months using the following questions:

**For in-patient care:** *HC202. Over the last 12 months, how many times you were admitted as patient to a hospital/long-term care facility for at least one night?*

**For out-patient care:** *HC302. In past 12 months, how many times did you receive healthcare or consultation from a healthcare provider (including home visits)?*

The elderly sample of the study (aged 60 and above) consists of 31, 902 individuals. Among them 2,557 reported having visited an inpatient health facility in the past year, 19,501 not visited any inpatient health facility, and 9,844 had missing information. Similarly, of the 31,902 elderly individuals, 17,585 visited an outpatient facility (including home visits), 4,175 did not visit any outpatient health facility and 10,142 had missing information.

For the current analysis on distance travelled, we included elderly individuals who had visited a health facility and reported the distance they travelled for the service. This information was collected using the following questions:

**For in-patient care:** *HC220. How many kilometers from your residence is the health care facility in which you were most recently admitted? Distance to health care facility: ________km*

**For out-patient care:** HC314. How many kilometers is the health care facility from your residence? Distance to medical facility: ____________km

For outpatient care, the question HC314 (distance travelled) was not asked of respondents who reported home visits. This information was collected using the following question:

*HC305. Which type of facility did you last visit to see that healthcare provider?*

**Public facility:**

1. Health post/sub centers

2. Primary health center

3. Community health center

4. District hospital/ Sub-district hospital

5. Government/tertiary hospital

6. Govt. AYUSH hospital

**Private facility:**

7. Private hospital/Nursing home

8. Private clinic (OPD based service)

9. NGO/Charity/Trust/Church-run hospital

10. Private AYUSH hospital Others:

11. Health camp

12. Mobile healthcare unit

13. Pharmacy/drugstore

14. ***Home visit***

15. Other, please specify _______

This resulted in a sample of 2,495 (out of 2,557) and 16,879 (out of 17,585) elderly individuals who visited inpatient and outpatient health facilities, respectively and reported the distance travelled. Based on these samples, the inpatient distance travelled ranged from 0 kilometers to 2000 kilometers and the outpatient distance ranges from 0 kilometers to 2100 kilometers. For the analysis, the distance was further categorised into five groups: 0-10 kilometers, 11-30 kilometers , 31-60 kilometers, 60-120 kilometers, and 120+ kilometers, to show the distribution (%) of elderly individuals travelling these distances to seek healthcare services. Additionally, the sample has been used to compute and report the arithmetic mean distance (in kilometers) travelled by elderly individuals for inpatient and outpatient visits.
